# Preclinical Autoimmune Disease: a Comparison of Rheumatoid Arthritis, Systemic Lupus Erythematosus, Multiple Sclerosis and Type 1 Diabetes

**DOI:** 10.3389/fimmu.2022.899372

**Published:** 2022-06-30

**Authors:** Giulia Frazzei, Ronald F. van Vollenhoven, Brigit A. de Jong, Sarah E. Siegelaar, Dirkjan van Schaardenburg

**Affiliations:** ^1^ Department of Rheumatology and Clinical Immunology, Amsterdam Rheumatology and Immunology Centre, Amsterdam University Medical Centers, University of Amsterdam, Amsterdam, Netherlands; ^2^ Department of Experimental Immunology, Amsterdam University Medical Centers, University of Amsterdam, Amsterdam, Netherlands; ^3^ Amsterdam Rheumatology Center, Amsterdam, Netherlands; ^4^ Department of Neurology, MS Center Amsterdam, Amsterdam University Medical Center (UMC), Vrije Universiteit Amsterdam, Amsterdam Neuroscience, Amsterdam, Netherlands; ^5^ Department of Endocrinology and Metabolism, Amsterdam University Medical Centers, University of Amsterdam, Amsterdam, Netherlands; ^6^ Amsterdam Rheumatology and Immunology Center, Reade, Amsterdam, Netherlands

**Keywords:** rheumatoid arthritis (RA), systemic lupus erythematosus (SLE), multiple sclerosis (MS), type 1 diabetes (T1D), prodromal phase, genetic risk factors, environmental risk factors, pathophysiological process

## Abstract

The preclinical phase of autoimmune disorders is characterized by an initial asymptomatic phase of varying length followed by nonspecific signs and symptoms. A variety of autoimmune and inflammatory manifestations can be present and tend to increase in the last months to years before a clinical diagnosis can be made. The phenotype of an autoimmune disease depends on the involved organs, the underlying genetic susceptibility and pathophysiological processes. There are different as well as shared genetic or environmental risk factors and pathophysiological mechanisms between separate diseases. To shed more light on this, in this narrative review we compare the preclinical disease course of four important autoimmune diseases with distinct phenotypes: rheumatoid arthritis (RA), Systemic Lupus Erythematosus (SLE), multiple sclerosis (MS) and type 1 diabetes (T1D). In general, we observed some notable similarities such as a North-South gradient of decreasing prevalence, a female preponderance (except for T1D), major genetic risk factors at the HLA level, partly overlapping cytokine profiles and lifestyle risk factors such as obesity, smoking and stress. The latter risk factors are known to produce a state of chronic systemic low grade inflammation. A central characteristic of all four diseases is an on average lengthy prodromal phase with no or minor symptoms which can last many years, suggesting a gradually evolving interaction between the genetic profile and the environment. Part of the abnormalities may be present in unaffected family members, and autoimmune diseases can also cluster in families. In conclusion, a promising strategy for prevention of autoimmune diseases might be to address adverse life style factors by public health measures at the population level.

## Introduction

Autoimmune disorders are diseases in which the immune system recognizes and reacts against self-antigens. Clinical onset is often preceded by low grade inflammation (1), disease-specific autoimmune features and nonspecific signs and symptoms. Little is known about similarities and differences between these diseases concerning the time course, nature and extent of inflammatory or autoimmune events. In the last 20 years, the number of individuals affected by autoimmune diseases has increased, especially in the more economically developed countries (2–4). Various mechanisms have been proposed to explain the increased incidence and prevalence, some of which might be shared between different autoimmune diseases.

Individuals in the pre-clinical phase have an initial asymptomatic phase of varying length in which the immune system is activated and the autoimmune process is started. Oftentimes, this phase is followed by nonspecific signs and symptoms and it might take years for the disease to manifest itself. To shed more light on this matter, we here compare the preclinical disease course of a selection of four important autoimmune diseases: rheumatoid arthritis (RA), Systemic Lupus Erythematosus (SLE), multiple sclerosis (MS) and type 1 diabetes (T1D). Although these are clinically distinct diseases, involving different autoimmune reactions and target organs, in some cases they appear to share certain genetic and environmental risk factors as well as pathophysiological mechanisms (5, 6). These insights may help to design strategies to prevent the development or progression of autoimmune diseases in general.

This review describes the evolution of disease manifestations from the pre-clinical phase up to clinical disease when the diagnosis can be made. We thereby focus on similarities and differences between the selected diseases rather than provide an in-depth review per disease. The review is not intended to give an overview of intervention studies in the at-risk phase, since these are discussed in another article of the present issue. The data were collected from literature *via* PubMed and Medline (**Box 1**).

## Overview of Autoimmune Diseases

**Figure 1:**
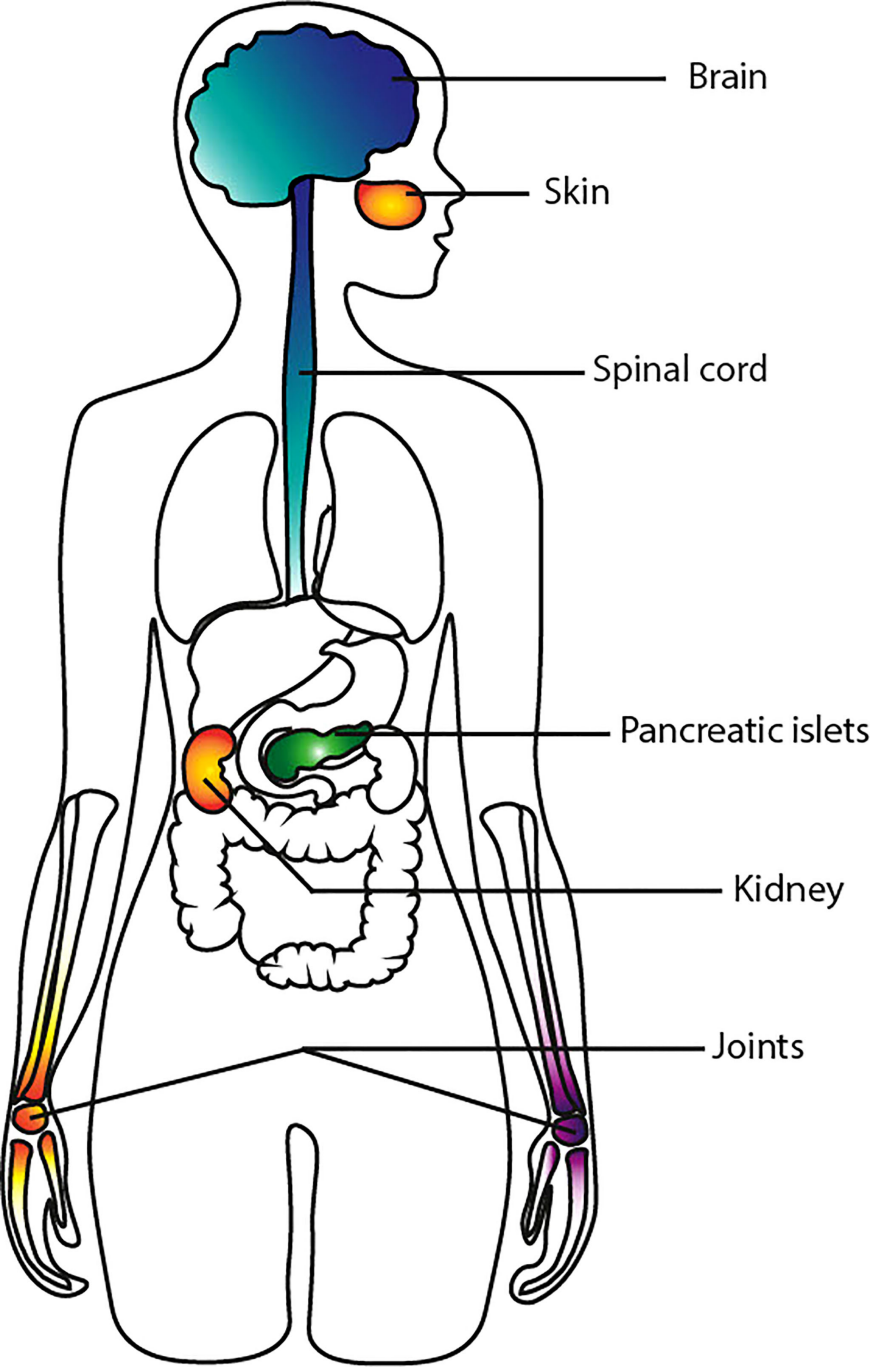
Primary site of onset. This illustration shows the primary site of initiation of the autoimmune process in RA, SLE, MS, and T1D. RA is represented in purple, SLE in orange, MS in blue and T1D in green.

RA, SLE, MS and T1D are autoimmune diseases which affect specific organs (**Figure 1**), with a later shift towards systemic compromise due to complications and comorbidity. They may also be associated to varying degrees with systemic inflammation. In the pre-clinical stage of autoimmune diseases, individuals have risk factors, both genetic and environmental, which predispose them to the disease. In the next paragraphs, we’ll present an overview of those risk factors, and how they might be similar or differ between diseases.

### Rheumatoid Arthritis

RA is an organ-specific autoimmune disease mainly characterized by a symmetrical peripheral polyarthritis, in which systemic inflammation and other manifestations may also be associated. RA affects 0,5-1% of the population worldwide, with a higher prevalence in regions at greater distance from the equator (7, 8). RA is seen as an autoimmune disease due to the presence of autoantibodies such as rheumatoid factor (RF) and anti-citrullinated protein antibodies (ACPA) in the majority of cases, which is then associated with a more severe disease course (9, 10).

In RA there is a familial clustering of disease, and a family history of RA increases the risk of disease by three to ten times (11, 12). This indicates an important role of genetic factors in disease risk. Indeed, more than 100 loci have been found associated to RA (13). The most relevant alleles are the ‘shared epitope’ (SE) at the *HLA-DRB1* locus and Protein tyrosine phosphatase (*PTPN22*) (9, 14, 15). *HLA-DRB1* codes for a cell surface molecule with a peptide-binding groove that has high affinity towards citrullinated proteins (16, 17). Other important genetic factors in RA are *CLTA4* and *PADI4*, involved in the immune system regulation and post-translational conversion of arginine to citrulline residues, respectively (9, 18). RA has also higher incidence in women, with a female-to-male ration of 2-3:1 (19).

Several environmental factors contribute to the risk of RA. The most prominent one is smoking (20–22), which as a risk factor interacts with SE (17, 23, 24). The increased risk of RA associated with smoking requires long term exposure to manifest, but moderate cigarette consumption is enough to affect disease risk and individuals will have a high RA risk even years after smoking cessation (22, 25). Similarly, other airway irritants such as silica and textile dust exposure are associated with increased risk of RA (21, 26, 27). Additional lifestyle behavior, such as lack of exercise, stress, and an unhealthy diet, all contribute to increasing the risk of developing RA (28–33). Studies that have shown an association between high birth weight and RA suggest that even environmental exposures *in utero* may contribute to the risk for RA (34, 35).

The average age of onset of clinically manifest RA is around 50 years old. The onset is preceded in many cases by a preclinical phase characterized by activation of the immune system and production of autoantibodies. Circulating autoantibodies together with low level inflammation as measured by high sensitive CRP are found on average 5 years before the onset of symptoms (36, 37). In one prediction model (38) using demographic, clinical and serological characteristics, individuals in the highest risk category had an 80% probability of developing RA within 5 years. Immune cell recruitment is usually followed by non-specific musculoskeletal symptoms and fatigue (39). Moreover, pain and transient swelling of the joints are common symptoms in at risk individuals (30-60% of seropositive individuals) (40).

In the years preceding symptoms, the autoantibody response broadens to include more and more ACPA specificities (37, 41–43), anti-acetylated peptide antibodies (AAPA), anti-carbamylated (anti-CarP), and RF, which is referred to as epitope spreading (44–48). In the months before clinical onset, ACPA additionally undergo glycosylation changes both of the Fc part and the Fab part of the ACPA-IgG molecule, leading to a more pro-inflammatory phenotype (49–51). It has been hypothesized that environmental exposure related to the respiratory tract (smoking, dust, respiratory infections) might be involved in antibody production and disease pathogenesis (21, 26, 27). Those environmental triggers could cause low-level inflammation of the lung mucosae, leading to protein citrullination (23, 52).

In patients with early RA, studies of low level inflammation at mucosal sites such as the gums and the lungs have revealed that these inflammatory lesions can be involved in local ACPA production. Transfer of ACPA to the joints may then be one mechanism that incites inflammation at the joint level. Soluble factors also regulate the immune response, and both pro-inflammatory and anti-inflammatory cytokine levels are altered in the preclinical phase (53). Markers of inflammation, such as C-Reactive Protein (CRP), are also increased up to 5 years before RA onset and positively correlate with antibody levels. Individuals with both elevated CRP and autoantibodies are more likely to develop RA (36, 37, 54). Increased plasma levels of polyunsaturated fatty acid-derived lipid mediators such as 5-Hydroxyeicosatetraenoic acid (5-HETE) are seen in ACPA positive individuals who later develop inflammatory arthritis (IA), further increasing the risk and pointing to a low omega3 fatty acid status. Cytokines associated with 5-HETE, such as IL-1β, IL-6, IL-8, and TNF, are also altered in preclinical RA individuals (55, 56).

In the phase with vague symptoms such as stiffness or arthralgia, inflammation of the joints can sometimes already be detected by imaging modalities as ultrasound, MRI and PET scan. In particular, subclinical inflammation of the joints detected by MRI predicted RA onset by a few months (57–60). At the time RA is diagnosed, patients usually have symmetrical polyarthritis in the hands and/or feet, which when left untreated can progress to joint destruction (61, 62). In established RA there is an overlap with other diseases, or comorbidity, including cardiovascular disease, chronic lung disease and periodontitis (63, 64).

### Systemic Lupus Erythematosus

SLE is characterized by a great variety of clinical manifestations, including inflammatory skin lesions, arthritis, pleurisy and pericarditis, inflammation in the internal organs, involvement of the central or peripheral nervous system, hematological manifestations, and others. The disease course is highly variable, some patients experiencing long periods of remission (the absence of disease manifestations), but many more experiencing frequent flares of disease activity and/or chronic symptoms. For many patients, the general feeling of illness, accompanied by fatigue, lassitude, and minor cognitive difficulties is the most burdensome feature of the disease.

SLE is an uncommon disease with wide geographic variation in distribution, with high frequency in North America; SLE also has higher frequency in the Afro-American population compared to Caucasians, which may be due to both genetic and environmental differences (65). There is a genetic component of disease, with concordance in monozygotic twins of 24-35% as compared to 2-5% in dizygotic twins (66). In the Caucasian population, HLA-DRB1*1501 and HLA-DRB1*0301 are associated with a 2-to-3 fold increase risk of SLE (67, 68). Other genes strongly associated with SLE are those coding for the complement system and the Fc-γ receptor (FcγR), all of which have a role in immune regulation (69–71). Genes that are involved in the IFN pathway, such as Interferon Regulatory Factor 8 (*IRF8*), *IFIH1*, Toll-like receptor 7 (*TLR7*), and Tyrosine Kinase 2 (*TYK2*) are also risk loci for the disease (72, 73). SLE also affects women much more frequently than men (female-to-male ratio 9:1) (65, 74, 75).

Smoking is a risk factor for SLE and is associated with higher anti-double strand (anti-dsDNA) antibody production (76). In the Nurses’ Health Study, nurses that smoked had a 67% increased risk of developing SLE compared to non-smokers, although the intensity of smoking did not influence disease risk. This association is time-sensitive, and the increased SLE risk persists for up to five years after quitting (77). It has been suggested that vitamin D may have a role in SLE pathogenesis and progression, and vitamin D supplementation might ameliorate inflammatory and hemostatic markers, however, this is controversial (78, 79). Lack of sleep is also associated with the transition to SLE in one study (80).

SLE occurs at all ages but the peak incidence is in the 3^rd^ and 4^th^ decades of life, and men have a later peak incidence compared to women (81, 82). Studies of the evolution of SLE from a healthy state through a preclinical phase to full-blown disease are complicated by the fact that the diagnosis of SLE cannot be made until sufficient clinical manifestations have occurred, to give the clinician the confidence that the diagnosis is correct. Thus, it is quite common for individuals to experience some joint pains and skin lesions for several years without a diagnosis. But then, an episode of pleurisy and the discovery of antinuclear (ANA) and anti-dsDNA antibodies leads to the diagnosis of SLE. No serious observer can doubt that the earlier joint and skin symptoms were manifestations of the same disease *process*, yet it would not have been correct to make the diagnosis of SLE at that time. In some cases, intermediate disease categories are used, such as “incomplete lupus” or “undifferentiated connective tissue disease”, but lack of uniform definitions and the variety of clinical and laboratory manifestations that are seen have hampered further progress.

Thus, it has been challenging to investigate the pre-clinical phase of SLE. A landmark study by Arbuckle et al. found that the emergence of autoantibodies preceded the clinical disease by many years, and there seemed to be a strict order by which they manifest: the first antibodies to appear are ANA, antiphospholipid, anti-Ro (SS-A) and anti-La antibodies (SS-B), which manifest at the same time. Anti-Ro antibodies are detectable in the serum approximately four years before SLE clinical manifestations. Subsequently, anti-dsDNA antibodies become manifest months before clinical diagnosis, followed by anti-Sm and anti-nuclear ribonucleoprotein (anti-RNP) antibodies, whose levels start increasing exponentially up to a year before diagnosis and are highest just before the disease is diagnosed (83, 84).

SLE also has alterations in levels of pro-inflammatory cytokines long before the onset of clinical signs and symptoms, with increased type I and II interferon (IFN-I and IFN-II), IL-5, IL-6, IL-17, and TNF (84, 85).

### Multiple Sclerosis

MS is an inflammatory demyelinating disorder of the central nervous system (CNS) with a presumed autoimmune pathogenesis. Several genetic, environmental and lifestyle risk factors are reported. A latitudinal gradient has been found, i.e. the farther away from the equator the frequency of MS increases. This latitudinal risk factor may reflect differences in UV radiation, sun exposure, vitamin D levels and epigenetic interactions. Migration from a higher to a lower latitude after puberty has an impact on disease risk: migrants retain its original risk (6, 86, 87). Genetic predisposition has a role in disease susceptibility, with 5% disease concordance in dizygotic twins that increases to 25% in monozygotic twins (87). HLA alleles exert the most common genetic risk factors, in particular the HLA-DRB1*1501 haplotype has been demonstrated to be the most significant genetic risk factor to develop MS (odds ratio approximately 3) (5, 88). More than 500 small nucleotide polymorphisms (SNPs) are associated with MS risk, involving mostly immune associated genes, such as IL-2 receptor subunit alpha *(IL2RA)*, *IL7R*, *CLEC16A* and *CD226* (5). MS is more frequent in women, with a female-to-male ratio of 2-3 (89).

Cigarette smoking contributes to the risk of MS, with a 50% higher risk in ever smokers compared to never smokers (86, 87). Two environmental factors that influence MS risk are vitamin D levels and Epstein-Barr virus (EBV) infection (6, 86, 87, 90, 91). Vitamin D deficiency in earliest stages of life is associated with increased risk of MS, while high sun exposure during childhood correlates with lower risk of disease (5, 6). In addition, the Nurses’ Health Study showed a 40% decrease risk of MS in women that had at least 400 international unit (IU) of vitamin D intake per day. Childhood obesity is associated with a higher risk to develop MS (86, 87, 92). Although the mechanism of action has not been fully elucidated yet, EBV infection seems to be a causative and necessary but not sufficient agent to develop MS (5, 86, 87, 90, 91). Recently, Lanz et al. demonstrated a high-affinity molecular mimicry between the glial cell adhesion molecule (GlialCAM) in the CNS and EBV nuclear antigen 1 (EBNA1). Considering that nearly 100% of MS patients has detectable anti-EBNA1 antibodies before clinical symptoms, it suggests that molecular mimicry may play a role in the pathophysiological mechanism to induce MS (93). Age of infection also influences disease risk, with 2-to-3 fold higher risk in individuals with EBV infection at later age (87).

In general patients are identified when they first manifest signs and symptoms characteristic for this disease (94). Most patients are diagnosed between age 20 and 40 year, however children and people of older age may also be diagnosed with MS (89). The clinical phase of MS is preceded by a latent period, in which a prodromal phase of MS can be identified (95). The prodromal phase can manifest 10-15 years before symptom onset, even up to 20 years in primary progressive MS (PP-MS). In this phase, an early set of sign and symptoms that predates classical MS symptoms start to manifest (96). A subclinical inflammation (SCIN) phase seems to be the first step of disease pathogenesis (94). While no formal biomarkers of the prodromal stage are available, the radiologically isolated syndrome (RIS) might be considered a neuroimaging biomarker (96, 97). In RIS, the CNS shows lesions similar to those identified in MS patients without clinical symptoms suggestive of MS, with areas of the brain and the spinal cord that show signs of damage and scarring (97).

Serum neurofilament light chain (sNfl) is indicative of ongoing neuraxonal degeneration, and can be used as a biomarker for neuronal injury. MS patients usually have high levels of sNfl that decrease after treatment with disease modifying therapies, and MS risk positively correlates with higher sNfl levels in a time-dependent manner, starting several years before MS (median of 6 years) (96, 98). In the earliest stages of disease, the adaptive immune system is mostly involved in pathogenesis, in particular with autoreactive T cells, B cells, and autoantibody production against myelin proteins (99, 100). T and B cell in spinal fluid are altered in prodromal MS, and present a pro-inflammatory cluster, with high percentage of expanded CD8+ T cells within the neuronal lesion (94).

MS can either manifest as episodes of inflammation with neurological symptoms followed by partial or total remission (relapsing remitting MS, RRMS, 85% of patients), or as a gradually progressive disease (PPMS). In time, RRMS may evolve into a progressive phase of the disease called secondary progressive MS (SPMS). Depending on the site of the lesion, patients may have different clinical pictures. Common presenting symptoms in RRMS are optic neuritis and ascending sensory symptoms, whereas PPMS in general presents with progressive motor impairment (88).

### Type 1 Diabetes

In T1D autoimmunity targets the beta-cells of the pancreas eventually resulting in absolute insulin deficiency. Similarly to the diseases mentioned above, T1D incidence is also affected by the latitudinal gradient and migration, with increased disease risk when populations move from low-incidence to high-incidence countries (6, 101). However, genes have a relevant role in disease risk, and relatives of T1D patients have a 15-20 times higher risk of developing T1D, rising from about 0,4% in the general population to 25-50% in monozygotic twins (102). Familial risk is mostly linked to HLA genes, and decreases to 1% in non-HLA genes (5). HLA are the most common alleles involved in T1D, but other relevant genetic risk factors include genes involved in the insulin and metabolism, as well as regulators of the immune response (5, 103).

In contrast with the other diseases discussed here, there is no demonstrated association between smoking and T1D (6), which might be explained more by the young age of patients at disease onset than by a true lack of a role of smoking in disease pathogenesis. Low physical activity, psychological stress and psychological trauma are associated with T1D risk (101). Vitamin D supplementation leads to lower autoantibody levels which may be beneficial in the early stages of disease (5, 15).

Diet may also influence T1D risk, as there is an increased risk in overweight children (101). Cow’s milk consumption is associated with islet autoimmunity (IA) and pancreatic beta cell destruction (15, 101). Other possible risk factors for T1D are viral infections, such as enterovirus, Coxsackie B viruses (CBVs), and respiratory viruses. Viral infections seem to correlate with incidence of islet autoimmunity (5, 101).

T1D present two peaks of incidence at 4-7 years old and – more commonly – 10-14 years of age (104, 105). T1D pathogenesis is characterized by three stages, two of which compose the preclinical phase. The first, asymptomatic stage involves immune recognition and activation with autoantibody production, initial beta cell destruction, but absence of dysglycaemia. In the second stage, progressive islet destruction and loss of beta cell mass leads to impaired insulin production and eventually dysglycaemia. Individuals in this stage are still asymptomatic (15), however, this stage evolves gradually. When approximately 80% of beta cell mass is destructed, glucose will rise and patients will become symptomatic. The percentage beta cell loss needed before symptoms arise decreases with age (106). The insulitis, persistent inflammation of pancreatic cells, is associated with functional impairment in the latest stages of preclinical disease (107). However, functional biochemical testing might already show impaired glucose tolerance.

Biomarkers of the T1D preclinical phase, and its progression towards clinical manifestation, are also the proinsulin to c-peptide (PI:C) ratio and reduced pancreatic volume. The first is indicative of beta cell stress, while the latter seems to correlate with reduced pancreatic islets and loss of exocrine volume. At-risk individuals, especially children younger than 10 years old, that progress to T1D have higher serum PI:C ratio than those who never progress to T1D. Moreover, FDR of T1D patients have reduced pancreatic volume compared to seronegative individuals, although it is still higher than patients with recent onset T1D (108, 109).

High levels of CD4+ and CD8+ T cells with specificity for beta cell autoantigens are now found in the islets of asymptomatic individuals. This antigen recognition might be mediated by B cell antigen presentation to T cells (107, 110). As said, autoantibodies are the first markers of disease. There are five main autoantibodies directed against insulin and islet cells. They precede clinical manifestations of T1D and are markers of beta-cell autoimmunity: autoantibodies against insulin (IAA), autoantibodies against insulinoma-associated antigen-2 (IA-2), autoantibodies against glutamic acid decarboxylase (GAD or GADA), autoantibodies against zinc-transporter 8 (ZnT8), and islet cell antibodies (ICA). The distribution of the different antibodies is age-related as IAA is the main antibody found in children, while GADA is most commonly found in young adults (111). Post-translation modification of insulin causes the formation of new epitopes that are recognized by autoantibodies involved in T1D pathogenesis (15, 103, 112, 113). The probability of diabetes development is dependent on the number of islet antibodies found in one person (114).

The symptomatic stage of T1D manifests as polyuria, polydipsia due to hyperglycemia, and eventually ketoacidosis caused by excessive lipolysis due to insulin deficiency and can only be treated with insulin replacement therapy (106).

## Comparison Between Diseases

The four diseases included in this review can affect a wide range of organs and tissues, that may be the initial site of an attack by the immune system. In line, the resulting pathology is diverse and one could easily conclude that the diseases have little in common. However, when one looks beyond the clinical manifestations to the genetic, environmental and behavioral determinants, it appears that apart from the differences there are also some notable similarities (**Table 1**). These include (in the majority of diseases) aspects such as a North-South gradient of decreasing prevalence (6, 86, 87, 101), a female preponderance (19, 89, 115), major genetic risk factors at the HLA level, partly overlapping cytokine profiles and lifestyle risk factors such as obesity, smoking and stress. Of note, T1D has predominance in males (116).

**Table 1:** Overview of selected major characteristics, risk factors, immunological and clinical features of four autoimmune diseases.

Variable		Disease			
		*RA*	*SLE*	*MS*	*T1D*
***Characteristics***		North-South declining gradientFamilial clusteringFemale-to-male ratio 2-3:1Average onset age 55 yrs	High frequency in North AmericaFamilial clusteringFemale-to-male ratio 9:1Average onset age 35 yrs	North-South gradientFamilial clusteringFemale-to-male ratio 2-3:1Average onset age 30 yrs	North-South gradientFamilial clusteringFemale-to-male ratio 1:1.8Average onset age 5 yrs (peak 1) or 12 yrs (peak 2)
***Genetic risk factors***	*HLA*	HLA-DRB1 (SE)	HLA-DRB1HLA-DQHLA-DR	HLA-DRB1HLA-DR3HLA-DR4HLA-DR6	HLA-DRB1HLA-DR4
	*Non-HLA*	PTPN22CLTA4PADI4	Complement system (C1q, C2, and C4)FcγR (FcγRI (CD64), FcγRII (CD32), and FcγRIII (CD16))MAVSIFN pathway (IFIH1, IRF5, TLR7, TYK2)	IL2RAIL7RCLEC16ACD226	INSPTPN22CTLA4SH2B3BACH2IL2RAIL7RCLEC16ACD226
***Environmental risk factors***		SmokingDustLack of exerciseObesityStress	SmokingVitamin D – controversial[Obesity][Lack of sleep]EBV infection	SmokingVitamin D/Lack of UV radiationObesityEBV infection	Vitamin DObesityInfectionsPsychological stress/traumaDiet – [cow’s milk]
***Preclinical immune system***		Autoantibodies (ACPA, RA, anti-CarP, AAPA)Cytokines (↑IL-1β, IL-2, IL-4, IL-6, IL-10, IL-17, TNF-α, IFN-γ)T cells (Th2, low Treg cells)	Autoantibodies (ANA, antiphospholipid, anti-Ro, anti-La, anti-dsDNA, anti-Sm, anti-RNP)Cytokines (↑IFN-γ, IL-5, IL-6, IL-17, TNF)	Antibodies against myelin proteinT cells (expanded CD8+ T cells, altered Treg cell function) B cells and plasma cells in CNS lesion	Autoantibodies (IAA, IA-2, GADA, anti-ZnT8, ICA)Complement (C4d increased in pancreas of seropositive individuals)T cells (CD4+ and CD8+ T cells)
***Early clinical manifestations***		Symmetrical polyarthritis	Skin lesionsArthritisCNS and peripheral nervous system inflammationInternal organ inflammationHematological manifestations	Neuronal inflammationMonocular visual lossSensory and motor limb symptoms	PolyuriaPolydipsiaHyperglycemia

This table summarizes selected major genetic and environmental risk factors, the involvement of the immune system in the preclinical stage, and early disease manifestations. In brackets [] are risk factors which association has been found weak, either for lack of evidence or for weakness of the association itself.

The North-South gradient may point to genetic differences, but can also be partly due to different climatic influences or dietary habit differences between more Northern and more Southern regions. Likewise, the observed female preponderance may be related to reproductive hormonal factors or alternatively to X-linked genetic factors. For both explanations, the available data do not fully explain the predominance of females (117). The importance of the environment is illustrated by the effect of migration, as an example children that move from Nordic countries to southern countries in younger years have the same prevalence of MS and T1D as is present in the new country (6, 86, 87). Moreover, an increased prevalence of RA was observed after migration from rural to urban areas in South Africa (118). A central characteristic remains the lengthy period of asymptomatic to undifferentiated disease which can cover many years, suggesting a gradually evolving interaction between the genetic profile and the environment. Differently from RA, SLE, and MS, T1D symptomatology is dependent on the amount of beta cell destruction, with residual hormonal function preceding symptoms (106).

When we thus suppose there may be a partly shared pathophysiology between the four diseases, one might expect this to become apparent in a clustering of diseases in the same individual. RA and SLE can indeed occur together, a situation called “rhupus”, however, this is quite uncommon (119). MS and T1D also tend to have a lower overlap than expected by their prevalences, partially due to an opposite role of HLA haplotypes (5, 6). T1D on the other hand seems to predispose affected persons to develop RA, possibly due to shared genetic risk factors (120).

On the level of antibodies, we see that RA patients might express ANA antibodies, while SLE and T1D patients can also express RF and/or ACPA (121). Relatives of patients also have risk of developing autoimmunity, not necessarily the same as their affected relative. This might be due to both shared genetic and environmental risk factors, and a more pro-inflammatory state of the immune system. Indeed, the presence of autoantibodies and their related autoimmune disease predispose patients to manifest non-disease specific antibodies, in a process called poly-autoimmunity. Hence, the mechanisms involved and the timeline of autoantibody production are still not clear, but both genetic and environmental factors might be involved (121). Taken together, the four diseases show a modest overlap in occurrence but more overlap in autoimmune phenomena.

In this sections below, we look further into these overlapping aspects.

### Genetic Risk Factors

RA, SLE, MS, and T1D all have a genetic component, with familial clustering and higher risk of disease in first degree relatives of patients (FDR) (12, 66, 87, 102, 122). Several of these genetic risk factors are shared between the diseases, with similarities being most apparent between RA and SLE on one hand, and between MS and T1D on the other hand.

The most prominent genetic risk factors are alleles within the HLA class, in particular *HLA-DRB1*. HLA contributes to nearly 33% of RA risk (123).The HLA-associated risk in RA with an odds ratio of around 6 is almost entirely due to a small peptide sequence present in a number of *HLA-DRB1* haplotypes, the ‘shared epitope’ (124, 125). In the Caucasian population, *HLA-DRB1* alleles are associated with a 2-to-3 fold increase risk of SLE, however, this association has not been seen consistently in the Afro-American population (67, 68, 126). On the other hand, specific HLA haplotypes might have a protective role in MS and T1D, such as HLA-DRB1*01, HLA-DRB1*10, HLA-DRB1*11 and HLA-DRB1*14 (5). HLA-DRB1*04 is a risk factor in both RA and T1D (5, 24), while HLA-DRB1*1501/DQB1*0602 have an opposite effect in MS and T1D, with an increased risk for MS but a protective role in T1D (5, 87). However, several other SNPs of the HLA gene associated with T1D also seem to be associated with MS (5, 6). Both MS and T1D have an epistasis effect, with haplotype-specific interactions between alleles of different parental origins (5).

RA, SLE, and T1D also share non-HLA risk factors with other autoimmune diseases, such as celiac disease, psoriasis, and autoimmune thyroid disease (5, 127, 128). Moreover, loci on the chromosome 3 have a 16% relative contribution to the risk of RA (123). A large number of non-HLA genes involved in autoimmune diseases are interlinked in a network that regulates interferon signaling and dendritic cell (DC) and T cell function. The tyrosine kinase cell-surface receptor *FLT3*, also known as CD135, is expressed on DC, and lymphoid and myeloid progenitors, and is involved in the regulation of monocyte and DC maturation. A specific intron variation in *FLT3* causes the production of a truncated protein, with decreased levels of FLT3 receptor and increased circulating FLT3 ligand, which could lead to autoimmunity. *FLT3* is associated with increased risk of RA, SLE, and T1D, and high levels of FLT3 ligand are found in both serum and synovial fluid of inflamed joints of RA patients (127).

Both RA and SLE show an association between disease risk and genes that are involved in type I interferon production, signaling, and response, such as *IRF5*, Interleukin 1 receptor associated kinase 1 (*IRAK1*), and Signal transducer and activator of promoter 4 (*STAT4*) (9, 14). In SLE, in presence of anti-RNA binding proteins (RBP) and anti-dsDNA antibodies, *IRF5* is associated with higher levels of circulating type I interferon activity. Additionally, *IRF5* variants are associated with higher antibody production predisposition in healthy individuals, which could form immune complexes that activate innate immune cells through over activation of the toll-like receptor (14).

A SNP haplotype of the *STAT4* gene in the third intron is associated with both RA and SLE, with higher risk when this SNP is present in both alleles. *STAT4* is involved in the signaling of cytokines, such as IFN-I, IL-12, and IL-23, which promote differentiation of effector T cells towards a Th17 phenotype. However, *STAT4* has different roles in RA and SLE at least according to animal models: while in RA STAT4 deficiency in mice is protective, with inability of those mice to develop RA, in SLE STAT4-deficient mice have accelerated nephritis and higher mortality (129).

PTPN22, which codes for a protein involved in both T and B cell signaling, is also an important risk factor for RA, SLE, and T1D (9, 14, 15). In RA, *PTPN22* has a stronger association risk in male compared to female seropositive individuals, and gene carriers have an earlier onset of disease (9). *PTPN22* is one of the common non-HLA genes associated with T1D, together with *IL2RA*, which in turn is also associated with SLE and MS (15, 128). *IL2RA* is involved in lymphocyte activity regulation and confers a 28% and 33% increased risk of developing MS and T1D, respectively (6, 130).

Other non-HLA genetic factors involved in autoimmunity are small nucleotide polymorphisms (SNP) in immune associated genes, such as *IL7R*, *SH2B3*, *CTLA4*, *BACH2*, *CLEC16A* and *CD226*, and the latter are involved in both MS and T1D risk (5, 6, 9, 131–134). These SNP can either give a predisposition to both diseases, or be mutually exclusive, and some of the shared genetic risk factors between MS and T1D are directly associated with disease development (5, 6). Both in MS and T1D, the weight of the genetic predisposition in disease development depends on the family member affected by the disease, with a parent-of-origin effect (5, 135). In T1D, there is an higher risk associated with paternal heredity, while in MS the increased risk is associated with maternal heredity (5).

### Lifestyle and Environmental Factors

Although genetic factors play an important role in risk of autoimmunity, genetic predisposition is able to explain only up to 50% of the risk of developing RA and T1D, leaving half of the patients without any known genetic marker (122, 135). Numerous studies have investigated the role of environmental factors in disease development including lifestyle factors, comorbidities, external agent exposure and bacterial and viral infections (77, 136, 137).

Smoking is one of the most prominent environmental risk factors, and has a role in RA, SLE, and MS (20, 77, 87). Although no association has been described between smoking and T1D, this is more likely due to the young age of T1D onset. Smoking causes citrulline autoimmunity in the lung in genetically susceptible individuals (24, 52, 138) and also triggers the production of RF (20, 22, 23), explaining an association between smoking and seropositive RA. In SLE, smoking is a risk factor for anti-dsDNA production (77), while in MS smoking induces an increased axonal demyelination and disruption of the blood-brain barrier, in parallel with an immunomodulatory effect mediated by increasing both nitric oxide levels and its metabolites (87). Both in RA and MS, but not in SLE, smoking has a dose-response relation with disease risk (22, 25, 86). After smoking cessation, the increased risk for RA and SLE remains present for several years (22, 25).

Occupational exposure seems also to be a risk factor for autoimmune diseases; silica and other inorganic dust exposure have been reported to increase the risk of RA and SLE (27, 139, 140). However, these associations are not as strong as for smoking.

Additional lifestyle factors are exercise, alcohol consumption, diet and body mass index (BMI). Exercise and moderate alcohol consumption have been associated with decreased risk of RA and SLE (33, 141), while obesity is associated with higher risk of RA, SLE, MS and T1D (76, 141–145). In persons at risk for RA, the combination of obesity and smoking seems to synergistically increase the risk of RA (146). In the Nurses’ Health Study, overweight and obese women had higher risk of developing RA, MS, and T1D (86, 147, 148). Similarly, being overweight is associated with higher risk of T1D (101). Consequently, dietary factors may be expected to play a role in disease risk. The overall dietary quality influences the risk for RA, amounting to a 40% decrease in risk for seropositive RA in women in the highest versus the lowest quartile of dietary quality (32). As for MS, a highly enriched fish diet seems to be protective; populations in Northern countries with a diet high in fish and fish oils show a similar MS incidence to those in lower-latitude countries (86, 87). In the case of T1D, cow’s milk has been suggested to trigger an autoimmune response in genetically at-risk individuals that leads to the destruction of pancreatic beta cells (15, 101). This correlation has been also found in the Diabetes Autoimmunity Study in the Young (DAISY), in which children with low and moderate genetic risk that had higher cow’s milk intake also had higher risk of islet autoimmunity (IA) (149).

Either chronic stress or the presence or post-traumatic stress disorder (PTSD) have both been related to the subsequent occurrence of autoimmune diseases (28). In a study covering the whole population of Sweden, a diagnosis of a stress-related disorder increased the risk of any autoimmune disease by 50% in the whole period of 35 years thereafter, including the diseases discussed here. Furthermore, a large study on US veterans of the Iraq war showed a doubled risk of RA, SLE and MS in individuals affected by PTSD (150). An increased risk for RA was also found by the Nurses’ Health Study in nurses that had PTSD symptoms (151), and chronic stress and psychological trauma had also been suggested to be associated with T1D risk. At least for the effect of stress, this might be due to higher levels of cortisol, inducing insulin resistance while also modulating the immune response (101).

It is important to consider that many of the associations mentioned above have a tendency to cluster within the population. Unhealthy diet, lack of physical activity, obesity, chronic stress, as well as environmental exposure, low socio-economic status and low income, all co-segregate, making it hard to identify if the causal association found by observational studies is caused by one specific factor or a combination of them.

Vitamin D levels have been suggested to influence disease severity in both MS and T1D in a seasonal way, with higher relapses in MS and diagnostic rate in T1D linked to vitamin D status (5). The mechanisms behind this association are not clear. However, 25-hydroxy vitamin D (25(OH)D) levels, which reflect vitamin D absorption by UV light exposure, inversely correlate with MS risk in white individuals (86, 87). 25(OH)D levels also inversely correlate with BMI, especially above 30, which might suggest an indirect mechanism of BMI as a risk factor (86). The onset of the first demyelinating event in at-risk-of-MS individuals correlates with both sun exposure and vitamin D levels. Sun exposure is measured by the degree of actin damage, which was lower at the time of onset of disease (87). While there is no correlation between T1D and 25(OH)D levels at birth, a birth-cohort study in Finland showed that 1 year of supplementation of dietary vitamin D, at a dose of 2000 IU daily was associated with a reduced risk of developing T1D in children. This might indicate a role of vitamin D in the pathogenesis of T1D between birth and early childhood (152, 153).

Another factor that may play a role in disease risk are viral infections. In RA there is no consistent evidence of infections involved in the pathogenesis. Epstein-Barr (EBV) infection has been suggested to increase the risk of SLE (154, 155) and is a major environmental risk factor for MS development (91, 96). While individuals with elevated immunoglobulin levels against EBV have a 2-fold increased risk of developing MS, EBV seronegative individuals have a disease risk near zero. Moreover, this mechanism seems to be specific to EBV, since cytomegalovirus infection does not influence MS risk, suggesting that EBV infection may be partially necessary for MS onset (5, 86, 96, 156). It has been postulated that EBV infection either increases activation and expansion of T and B cells, or is responsible for B cell immortalization, in particular of B cells that produce antibodies against EBV, leading to antigen presentation to pathogenic T cells (87).

While the association between MS and EBV infection is strong, the role of infections inT1D pathogenesis is not yet well defined. The Diabetes Prediction and Prevention (DIPP) study demonstrated a correlation between first autoantibody appearance and enterovirus infection, and serological studies suggest a link between Coxsackie B virus, in particular CBV4 serotype, and T1D. Moreover, the Teddy study described a possible correlation between respiratory infections, with a common peak between 6 and 9 months of age, and increased risk of islet autoimmunity, which follow a similar trend (15, 157). In summary, there is evidence for a role of viral infections in the pathogenesis mainly of MS and T1D, with a very specific role of EBV in MS.

### Activation of the Immune System

**Figure 2:**
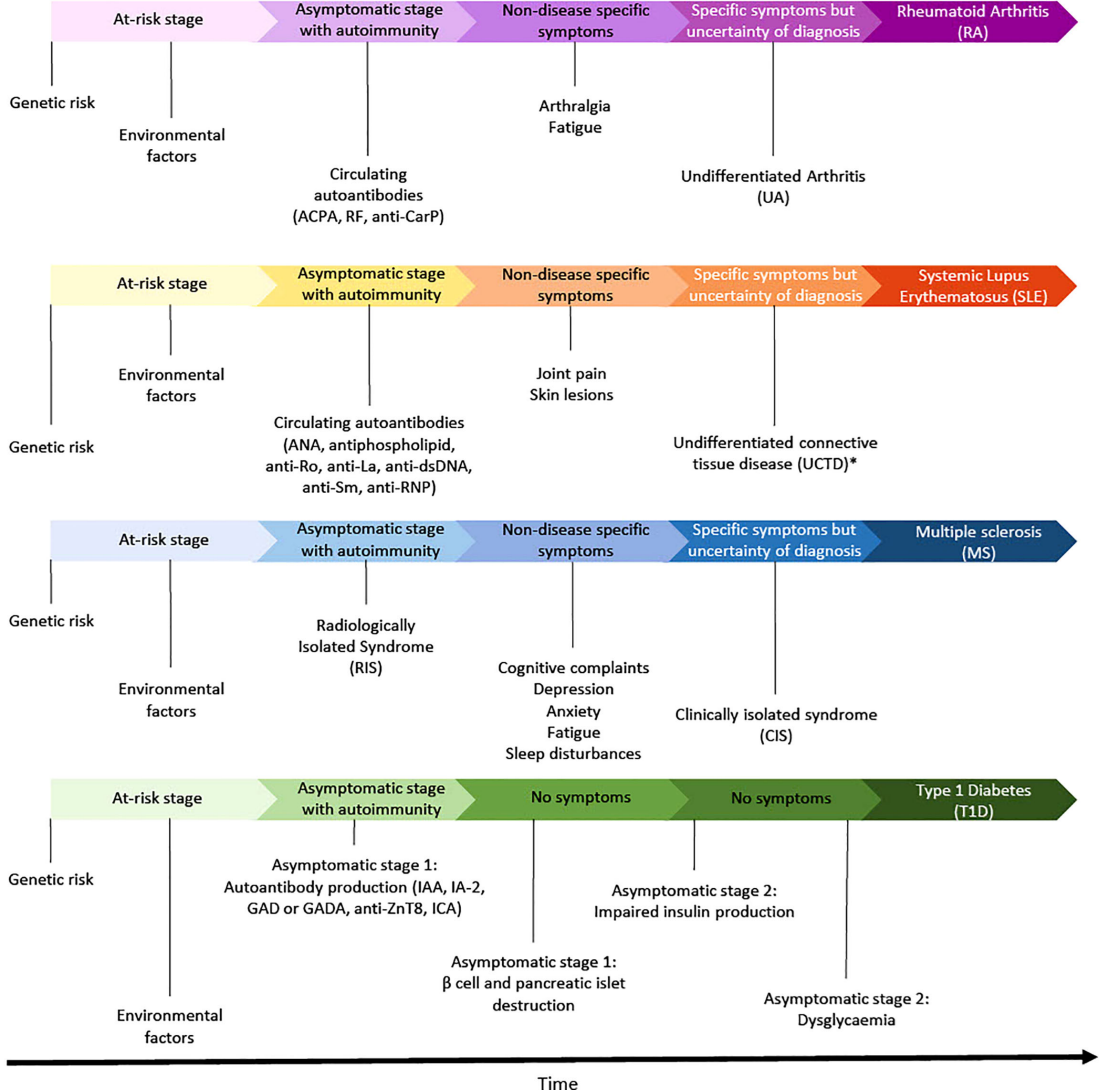
This illustration shows an overview of the transition from at-risk to disease diagnosis. In purple is represented RA, in orange SLE, in blue MS, and in green T1D. *Also known as “incomplete Lupus”. ACPA, Anti-citrullinated protein antibody; RF, Rheumatoid factor; anti-CarP, anti-carbamylated; ANA, antinuclear antibody; anti-dsDNA, anti-double strand DNA; anti-RNP, anti-nuclear ribonucleoprotein; IAA, autoantibodies against insulin; IA-2, autoantibodies against insulinoma-associated antigen-2; GAD or GADA, autoantibodies against glutamic acid decarboxylase; anti-ZnT8, autoantibodies against zinc-transporter 8; ICA, islet cell antibodies.

RA, SLE, MS, and T1D all have a latent phase that precedes formal clinical diagnosis (**Figure 2**). The length of this phase can vary between diseases and within individuals at risk for the same disease, but a common feature is the activation of the immune system, which is visible to a varying degree in the different diseases and precedes the onset of symptoms.

#### Humoral Immunity

As described above, the majority of RA patients is seropositive, and these antibodies develop over many years before the clinical disease, with increasing concentrations as well as specificities (59, 158). In particular ACPA are thought to be involved in the development of synovitis and bony erosions.

In contrast, in SLE autoantibodies are uniformly found in all patients. This is in part due to the conceptions and definitions used for making the diagnosis of SLE in clinical practice, codified by the recent EULAR/ACR classification criteria for SLE where the presence of ANA is required (159).

Some autoantibodies in SLE play an important role in the pathogenesis; this is most convincing for anti-DNA antibodies. Furthermore, there is a strong association between the combination of multiple antibodies, such as anti-dsDNA and anti-C1q, decreased complement levels, and lupus nephritis (LN). The most reproducible autoantibodies for diagnostic purposes are those reflecting renal involvement (160, 161).

So far, in MS no specific autoantibody has been found, however, autoantibodies against several CNS cells have been reported in this disease (162, 163).

As noted above, also persons at risk for T1D can develop several types of autoantibodies. There is a combination effect of multiple antibodies, with 70% risk of disease in children with multiple (three or four) circulating antibodies. Young children preferably develop IAA, while GAD autoantibodies are most commonly found in teenagers. However, the conversion from single to multiple antibodies can be slow (111, 114, 164–166).

Nearly 60% of children with single autoantibodies will lose antibody production over time and convert to seronegative (15). This mechanism is unique to T1D and differs from RA and SLE.

Of note, FDR of RA, SLE, and T1D patients may have autoantibodies detectable in their serum in absence of any signs or symptoms of disease (83, 121).

#### Cellular Immunity

The cellular component of the immune system also has an active role in disease pathogenesis. In preclinical RA, ACPA+ individuals have decreased T regulatory (Treg) cell levels and a shift of CD4+ T cells towards pro-inflammatory subsets, in particular T helper (Th) 2 cells (53, 167). In SLE, numerous abnormalities of cellular immunity have been described (168) but it has been difficult to determine whether these are necessary elements of the pathophysiology of the disease itself or the consequences of long-standing inflammation or of the treatments used to control it. In established MS altered activity and levels of Treg cells and predominance of CD8+ T cells are found within the neuronal lesion (94, 169, 170). In the prodromal phase of MS, the frequency of expanded CD8+ T cells within the CNS increases, and cells show alterations of their markers towards a more pro-inflammatory phenotype (94). Autoreactive T cells target the myelin in MS and the pancreatic islets autoantigens in T1D patients and T1D relatives, respectively (171). In the asymptomatic phase of T1D, the high levels of CD4+ and CD8+ T cells that are specific for beta cell autoantigens cause persistent inflammation of the pancreatic islet, called insulitis. This antigen recognition might be mediated by B cell antigen presentation to T cells (107, 110).

The B cell component is also altered in individuals at risk for RA, who have higher levels of IgA plasmablasts than the general population (172). The importance of the B cell component in the evolution from at-risk individuals to RA has also been demonstrated in the PRAIRI study, a clinical trial in ACPA-positive at-risk individuals, in which B cell depletion through a single dose of rituximab significantly delayed disease onset compared to placebo (173). In SLE, patients have decreased levels of CD27-IgD-IgM B cells, which represent an activated and auto-reactive state (174). Expanded B cells are also found in the neuronal lesions of prodromal MS individuals, where they correlate with oligoclonal immunoglobulin bands (94). Moreover, B cells also have a role in the pathogenesis of T1D, as demonstrated by B cell depletion after 1 year treatment with rituximab. Patients that received the treatment had reduced impairment of beta cell function compared to placebo, and required less insulin for disease management (175).

#### Soluble Factors

Soluble factors have a role in disease pathogenesis, inducing immune activation, recruitment, and regulation of the immune response. They are also responsible for direct pathogenic manifestations and can be used in some cases as biomarkers of disease progression. Soluble factors involve a variety of molecules, such as cytokines, complement and markers of inflammation.

Preclinical RA individuals on average have increased levels of both pro-inflammatory and anti-inflammatory cytokines, such as IL-1β, IL-2, IL-4, IL-6, IL-10, IL-12, TNF-α, and IFN-γ. Cytokine levels change over time, IL-4 and IL-14 levels being higher at the earliest stages of disease, and IL-17 levels increasing before disease onset and decreasing after RA becomes established (53, 176). Type I interferon (IFN-I) is detectable in the blood of both at-risk and established RA individuals, and has also a role in SLE initiation of SLE. While in RA there is higher production of IFNβ, in SLE there is abundance of circulating IFNα. Treatment of viral hepatitis with INF-α has been associated with *de novo* onset of SLE, the symptoms of which would improve after the treatment is stopped. Serum levels of IFN-I increase drastically one year before SLE onset, and circulating IFN-I is considered an hereditary risk factor for SLE (14). IFN-γ is also increased in SLE individuals more than 3.5 years before diagnosis and is associated with increased anti-RNA antibody production, inflammation, and transition from undifferentiated disease to connective tissue disease (84, 177). First-degree relatives of patients with MS have on average a more pro-inflammatory cytokine profile (higher TNFα, lower IL-10), this suggests that differences in cytokine profile may contribute to the pathogenesis of MS (178)

The complement system is involved in both SLE and T1D. The presence of C1q deficiency in at-risk SLE individuals, together with increased IgG : IgM anti-dsDNA ratio, may be indicative of disease development (179). C4d has been found to be increased in the pancreas of 25% of T1D patients, while in non-diabetic individuals this percentage decreases to 7% of T1D associated autoantibody positive and 2% of autoantibody negative individuals (180).

### Preclinical Signs and Symptoms

In RA, autoantibody production precedes the first disease manifestations by years (158). In time, the low lover inflammation and ACPA and/or RF titers increase, followed by non-specific musculoskeletal symptoms (39). Other common symptoms in pre-RA are arthralgia, fatigue, reduced mental health due to limited functionality and work absence, and non-articular manifestations, such as cardiovascular diseases (181, 182). More than 60% of seropositive individuals tend to have pain, stiffness and swelling of the joint, and nearly 30% had joint tenderness, even before RA onset (40). Another study showed an increased frequency of primary care visits for musculoskeletal symptoms, infections and comorbidities in the years prior to the diagnosis of IA (183).

As explained above, the identification of a preclinical stage of SLE and the diagnosis itself is complicated by the need of sufficient clinical manifestation and the time elapse that this entails. Individuals in this phase may experience joint or skin symptoms for several years, associated with ANA and anti-DNA antibody production.

During the prodromal phase of MS decreased cognitive performance, fatigue, pain, depression, anxiety, bowel, and bladder disorders are more often reported in the 5 years before the diagnosis of MS. Individuals in the prodromal phase are also more likely to seek healthcare and present health deterioration 5-10 years before the first clinical event (96, 98, 184). Nearly one third of RIS individuals develop MS-related neurological symptoms within 5 years. Age younger than 35 years old, male gender, thoracic or cervical spinal cord lesion, and the presence of oligoclonal bands in the cerebrospinal fluid are major predictors of RIS conversion to MS (98, 185).

The preclinical phase of T1D can be divided into two stages, with an initial stage of immune recognition and antibody production, beta cell and pancreatic destruction, followed by an exacerbation of islet destruction that leads to insulin production impairment and dysglycaemia (15). Functional tests are able to detect an impairment of insulin production and dysregulation of glucose metabolism in the preclinical phase, however specific signs or symptoms are only shown with manifest hyperglycemia, the clinical stage (15, 186, 187).

## Discussion

The necessarily incomplete overview of the preclinical phase of four distinct autoimmune diseases presented in this narrative review naturally highlights several differences in pathophysiology and clinical manifestations, but also shows that many of their etiologic and pathophysiological features actually overlap. The picture that emerges of these autoimmune diseases is that of a genetically determined increased sensitivity to breach immune tolerance to certain body parts, that is triggered under the influence of often multiple environmental factors during many years. Highly prevalent environmental factors such as smoking, obesity and stress are related to all four of these diseases and are known in general to produce a state of chronic systemic low grade inflammation (1). Thus, although the genetic basis and clinical features of the diseases are quite specific, the trigger for their manifestation in many cases is quite general.

The preclinical or prodromal phase of these diseases is characterized by nonspecific symptoms and in some cases more specific signs of autoimmunity at laboratory testing, which increase towards the onset of clinically manifest disease and subsequent diagnosis. Thus a high risk of future clinical disease can mostly be measured accurately only shortly, typically in the last year or so, before onset of clinical disease. Such a high risk of imminent disease then provides the setting in which preventive interventions with drug therapy could be tested, a situation resembling very early treatment of the same disease.

Attempts at prevention at an earlier stage would then involve interventions directed at life style factors. However, since it is difficult to identify individuals with an only slightly increased risk for autoimmune diseases, preventive efforts for autoimmune diseases would then become part of the public health domain. Indeed, increased public health or legislation actions to reduce smoking and obesity, as well as other unhealthy behaviors, while being completely nonspecific, could have a huge impact on the incidence and burden of not only the autoimmune diseases discussed here, but chronic non-communicable diseases in general. Meanwhile, physicians treating persons with increased risk of these diseases will have to await further advances in the prediction of clinical disease and in the (cost-)effectiveness of preventive therapy in high risk individuals.

## Author Contributions

All authors listed have made a substantial, direct, and intellectual contribution to the work, and approved it for publication.

## Funding

The project has received funding from the European Union’s Horizon 2020 research and innovation programme under the Marie Skłodowska-Curie grant agreement no. 847551 (GF).

## Conflict of Interest

The authors declare that the research was conducted in the absence of any commercial or financial relationships that could be construed as a potential conflict of interest.

Box 1 **Search strategy and selection – We searched MEDLINE for publications in English using the terms “rheumatoid arthritis”, “systemic lupus erythematosus”, “multiple sclerosis”, and “type 1 diabetes”, “risk factors”, “preclinical”, “prodromal”, “asymptomatic”, and MEDLINE subheadings. We selected articles based on our opinion of their scientific importance. We focused on original research articles, and selected reviews from highly authorative journals. We provide an overview of four autoimmune diseases, comparing their similarities and differences in their preclinical stage.**

## Publisher’s Note

All claims expressed in this article are solely those of the authors and do not necessarily represent those of their affiliated organizations, or those of the publisher, the editors and the reviewers. Any product that may be evaluated in this article, or claim that may be made by its manufacturer, is not guaranteed or endorsed by the publisher.
